# Targeted metabolomics reveals serum changes of amino acids in mild to moderate ischemic stroke and stroke mimics

**DOI:** 10.3389/fneur.2023.1153193

**Published:** 2023-04-14

**Authors:** Shuxin Tao, Xinxing Xiao, Xin Li, Fan Na, Guo Na, Shuang Wang, Pin Zhang, Fang Hao, Peiran Zhao, Dong Guo, Xuewu Liu, Dawei Yang

**Affiliations:** ^1^Department of Neurology, Liaocheng People’s Hospital, Liaocheng, Shandong, China; ^2^Department of Clinical Laboratory, Zibo Central Hospital, Zibo, Shandong, China; ^3^Zhong Yuan Academy of Biological Medicine, Liaocheng People’s Hospital, Liaocheng, China; ^4^Experimental Research Center, China Academy of Chinese Medical Sciences, Beijing, China; ^5^Department of Neurology, Qilu Hospital of Shandong University, Institute of Epilepsy, Shandong University, Jinan, Shandong, China

**Keywords:** ischemic stroke, stroke mimics, targeted metabolomics, amino acids, biomarker

## Abstract

**Background:**

The pathophysiological processes linked to an acute ischemic stroke (IS) can be reflected in the circulating metabolome. Amino acids (AAs) have been demonstrated to be one of the most significant metabolites that can undergo significant alteration after a stroke.

**Methods:**

We sought to identify the potential biomarkers for the early detection of IS using an extensive targeted technique for reliable quantification of 27 different AAs based on ultra-performance liquid chromatography tandem mass spectrometry (UPLC-MS/MS). A cohort with 216 participants was enrolled, including 70 mild to moderate ischemic stroke patients (National Institutes of Health Stroke Scale < 15, MB group), 76 stroke mimics (MM group) and 70 healthy controls (NC group).

**Results:**

It was found that upon comparing MB and MM to control patients, AAs shifts were detected *via* partial least squares discrimination analysis (PLS-DA) and pathway analysis. Interestingly, MB and MM exhibited similar AAs pattern. Moreover, ornithine, asparagine, valine, citrulline, and cysteine were identified for inclusion in a biomarker panel for early-stage stroke detection based upon an AUC of 0.968 (95% CI 0.924–0.998). Levels of ornithine were positively associated with infract volume, 3 months mRS score, and National Institutes of Health Stroke Scale (NIHSS) score in MB. In addition, a metabolites biomarker panel, including ornithine, taurine, phenylalanine, citrulline, cysteine, yielded an AUC of 0.99 (95% CI 0.966–1) which can be employed to effectively discriminate MM patients from control.

**Conclusion:**

Overall, alternations in serum AAs are characteristic metabolic features of MB and MM. AAs could serve as promising biomarkers for the early diagnosis of MB patients since mild to moderate IS patients were enrolled in the study. The metabolism of AAs can be considered as a key indicator for both the prevention and treatment of IS.

## Introduction

Stroke is still a leading cause of death as well as disability worldwide with 2.4 million new strokes cases annually diagnosed in China (1, 2). The majority of patients are affected with ischemic stroke (IS) that accounts for more than 80% of all strokes. Parenchymal ischemia or infarction has been detected by using various important imaging techniques such as computed tomography (CT), magnetic resonance image (MRI), ultrasonography, and angiography. However, inaccurate imaging might result in false or missed diagnosis, for example, routine diagnostics with multimodal computed tomography (CT) has been found to display limited capacity for distinguishing IS from stroke mimics (SMs) within the first hours after the stroke (3). The new imaging techniques such as diffusion-weighted MRI can achieve better diagnostic sensitivity in comparison to both conventional MRI and CT scan. However, its application is limited because of high cost, low availability and time-consuming procedures. Therefore, there is an urgent need to develop novel biomarker-based tests to rapidly and accurately diagnose and discriminate IS.

In numerous physiological and pathological processes, amino acids (AAs) play a key role as a class of metabolites. AAs are the building blocks for protein synthesis, which results in multifunctional proteins and derivatives including neurotransmitters, important enzymes, and precursor chemicals. The stability of amino acid metabolism is an important condition for normal body function, and the remarkable disorder caused by IS is part of the underlying mechanism of pathological changes in the brain. Recent research indicates that amino acids may have both beneficial and detrimental effects (4). For instance, glutamate is important in maintaining normal nerve cell signal transduction, which is beneficial to neuronal synaptic plasticity and stroke recovery (5). However, high glutamate levels can cause oxidative stress, inflammation, and endothelial damage (6). Branched chain amino acids (BCAA) levels were also found to be low in stroke patients when compared to normal controls, and lower BCAA levels in stroke patients were associated with a poor neurological outcome. However, some of reported studies about changes of AAs in IS have been found to be inconsistent. For example, the levels of BCAAs, including valine, leucine and isoleucine, were consistently observed to be decreased in patients with IS (7, 8). However, other prior studies have reported that higher levels of BCAAs could be related to an increased risk of future IS (9, 10). Confounding results were also reported for alanine, glutamine and proline (11–13). As a result, elucidating the effects of dysregulated amino acid levels after stroke need a new reliable and comprehensive strategy.Metabolomics is a new powerful tool that can effectively detect changes of small-molecule metabolites, such as amino acids, organic acids and lipids, in the biofluids of patients in real time situations. Metabolomics profiling can be both targeted and non-targeted. The untargeted approach is used for the simultaneous measurement of as many metabolites as possible and to find new compounds (14, 15). In targeted analysis, a relatively small number of known metabolites is profiled. It has a relatively greater specificity and sensitivity than an untargeted approach (16). As metabolomics can potentially identify several small-molecule metabolites that can effectively traverse the blood–brain barrier, metabolomics has exhibited greater advantages in comparison to both proteomics and transcriptomics during identification of novel biomarkers of IS. A further benefit of metabolomic research is the potential to discover novel connected metabolic pathways, which can significantly advance the knowledge of the pathogenic processes in IS and aids in the development of targeted treatments.

There are several prior studies using metabolomics that have revealed the possible changes in metabolites between patients with acute IS and healthy controls (HCs) (17–19). Interestingly, changes in the metabolite levels have been reported, including that of AAs, lipids, nucleotides, short peptides in the plasma, brain tissue, and cerebrospinal fluid (CSF) in both the murine Model and IS patients (20). These metabolite alterations typically fall into one of three primary pathophysiologic subcategories, including that of inflammation, oxidative stress, and excitotoxicity or neurotoxicity (7). However, these altered metabolites have to be selected to serve as biomarker, they should be reliable, possess adequate sensitivity, specificity and good pathophysiological correlation (7). According to growing evidence, AAs are the most frequently altered metabolites that have been identified in context with IS.. Therefore, the main goal of this study was to reveal the metabolic alterations in various AAs during IS and whether AAs or what kinds of AAs can discriminate IS from stroke mimics and healthy controls using a targeted metabolomics approach with quantitative amino acid profiling.

## Materials and methods

### Patients

Patients were hospitalized in the Liaocheng People’s Hospital between May 2021 and March 2022. The inclusion criteria selected were as follows: patients who presented an initial National Institutes of Health Stroke Scale (NIHSS) score from 1 to 14, aged ≥18 years, presentation ≤24 h after stroke onset. Exclusion criteria included: baseline head CT or MRI results showing a diagnosis of cerebral hemorrhage, abscess, vascular malformation, tumor, or another nonischemic cerebrovascular disease, or severe liver and kidney dysfunction. The blood sampling was performed upon hospital arrival and prior to routine diagnostic multimodal CT. The final diagnosis of IS was based on the presence of a diffusion weighted imaging (DWI). The patients with a DWI-negative lesion on MRI were classified as having stroke mimics. The TOAST classification was evaluated by two professional neurologists. The study was approved by the ethics committee of the hospital (Number: 2021003). In addition, written informed consents were obtained from all the participants.

### Clinical examination

The demographic and clinical data of all the participants were collected from the medical record. The Blood count, total cholesterol (TC), triglycerides (TG), high- density lipoprotein (HDL), low-density lipoprotein (LDL), and creatinine were measured in the Clinical Laboratory Departments in Liaocheng People’s Hospital and vascular risk factors (such as hypertension, hyperglycemia, and hyperlipidemia) were defined as described in the previous studies and were carefully inquired by clinical physician (21, 22). We also evaluated the National Institutes of Health Stroke Scale (NIHSS) at admission that were obtained at the same time from each patient. A score of 1–14 was classified as mild to moderate stroke (23). The modified Rankin Scale (mRS) score was evaluated at 90 days after stroke onset of all patients. A high signal intensity on the DWI sequence was used to indicate acute cerebral infarction. On DWI sequences, infarct regions were manually identified. Slice thickness and infarct area in each slice were multiplied to determine infarct volumes. 48 h following the onset of the symptoms, 1.5 Tesla scanners with 6 mm slice spacing were used for the MRI scans. A total of 70 HCs with age and sex matched with the IS group were recruited from the physical examination Center in the same hospital. Finally, 70 mild to moderate IS patients (NIHSS<15, MB group), 76 stroke mimics (MM group) and 70 healthy controls (NC group) were enrolled in current study.

### Sample preparation and UPLC MS/MS analysis

The targeted metabolomics analyses of AAs in the serum samples were performed by combining 4 μl serum and 991 μl of solution (1.7 mM ammonium formate in 85% acetonitrile containing 0.1% formic acid) and 5 μl of IS (phenylalanine-d5, 100 ng/ml). This mixture was vortexed for 1 min. After the centrifugation (12,000 rpm for 5 min at 4°C), 5 μl of the supernatant was directly analyzed by UPLC/MS–MS. The LC–MS/MS system was made up of a Waters ACQUITY UPLC system coupled to a Waters (Milford, MA, USA) Xevo TQ-S triple quadrupole MS with flow through-needle sample management, cooling autosampler, temperature-controlled column oven, degasser, and binary pump. The detailed chromatographic separations and MS conditions can be seen in method part in supplementary materials.

### Data analysis

A web-based analytical pipeline Metaboanalyst (24) was used for the multivariate statistical analyses of metabolomics data, including the partial least squares discriminant analysis (PLS-DA), biomarker analyses, and pathway analyses. Benjamini-Hochberg false discovery rate (FDR) adjustment for Student’s t-test was performed on the MetaboAnalyst. PLS-DA was conducted to visualize the possible global metabolic difference of individuals between controls, MB and MM groups. To validate the PLS-DA model, different permutation tests were performed to evaluate PLS-DA model reliability (n = 100) and R2 and Q2 were utilized to assess its quality. Biomarker analysis was used to obtain the receiver operating characteristic (ROC) curve-based approach for identifying the potential biomarkers and evaluating their performance. Pathway analysis, including pathway modules and pathway topology analyses, was employed to identify the various key biological pathways linked to the observed changes in metabolites.

Linear regression model analyzed the correlation between infarct volume, NIHSS score, 3 months mRS score and metabolites using SPSS Statistics 25.0 software, and the difference in *p* < 0.05 was statistically significant.

## Results

### Baseline characteristics of MB patients

70 mild to moderate ischemic stroke patients (NIHSS<15, MB group), 76 stroke mimics (MM group) and 70 healthy controls (NC group) were enrolled in the current study. There were no significant differences observed in age and sex among MB, MM and NC. We found that hyperlipidemia, coronary heart disease (CHD) and total cholesterol were significantly changed in MB, MM and NC (p<0.05). Additionally, other related clinical traits have been summarized in Table 1 by the form of mean ± standard deviation (SD).

**Table 1:** Clinical and demographic characteristics of patients.

	MM	NC	*P*	MB	NC	*P*
number	76	70		70	70	
Anthropometric characteristics						
age	65.33 ± 9.94	63.04 ± 7.03	0.109	64.80 ± 9.17	63.04 ± 7.03	0.206
Gender (M/F)	46/30	38/32	0.446	49/21	38/32	0.055
weight	69.50 ± 12.27	69.57 ± 12.39	0.975	69.03 ± 9.95	69.57 ± 12.39	0.780
History *n*(%)						
smoking	29(38%)	17(24%)	0.071	22(31%)	17(24%)	0.346
drinking	27(36%)	23(33%)	0.734	22(31%)	23(33%)	0.856
hypertension	46(61%)	33(47%)	0.105	41(59%)	33(47%)	0.176
hyperlipidemia	9(12%)	21(30%)	0.001	2(3%)	21(30%)	0.000
hyperglycemia	21(28%)	21(30%)	0.752	21(30%)	21(30%)	1.000
CHD	20(26%)	3(4%)	0.000	19(27%)	3(4%)	0.005
artial fibrillation	3(4%)	2(3%)	1.000	4(6%)	2(3%)	0.676
myocardial infarction	0(0%)	—		1(1%)	—	
family history	4(5%)	—		6(9%)	—	
Blood parameters						
serum glucose (mmoL/L)	6.02 ± 1.97	6.10 ± 1.06	0.769	6.76 ± 2.46	6.10 ± 1.06	0.039
total cholesterol (mmoL/L)	4.46 ± 1.00	5.07 ± 1.04	0.001	4.57 ± 1.09	5.07 ± 1.04	0.007
Triglyceride (mmoL/L)	1.62 ± 0.88	1.31 ± 1.18	0.080	1.48 ± 1.39	1.31 ± 1.18	0.433
LDL-C (mmoL/L)	2.75 ± 0.71	2.68 ± 0.88	0.623	2.77 ± 0.86	2.68 ± 0.88	0.547
Creatinine (μmoL/L)	58.93 ± 16.12	63.57 ± 15.71	0.085	64.98 ± 19.39	63.57 ± 15.71	0.638
infraction volumes	—	—	—	4.77 ± 7.64	—	—
NIHSS score	—	—	—	5.59 ± 3.20	—	—

### Targeted metabolite profiling of mild to moderate ischemic stroke patients

In this study, an LC/MS-based targeted metabolomics approach was used to quantify 27 different AAs. The quality control samples (QC) were prepared and regularly analyzed with the serum samples to ensure result reproducibility, with peak area RSD for these QC samples being <15% consistent with the reliability of the analytical methods used in this metabolomics study. The precursor ions, product ions, and collisions for 27 AAs have been shown in Table 2.

**Table 2:** Mass spectral data of the 27 target amino acids.

Ion mode	Compounds	Precursor (m/z)	Product (m/z)	CV(V)	CE(V)
ESI(+)	Isoleucine	132.06	86.09	20.00	12.00
ESI(+)	Leucine	132.06	86.09	18.00	10.00
ESI(+)	β-Alanine	90.02	30.03	24.00	10.00
ESI(+)	Phenylalanine	166.11	120.10	20.00	12.00
ESI(+)	γ-Aminobutyric acid	104.03	86.88	26.00	10.00
ESI(+)	Tryptophan	205.06	188.06	36.00	10.00
ESI(+)	Methionine	150.02	56.11	28.00	14.00
ESI(+)	Valine	118.05	72.00	26.00	10.00
ESI(+)	Proline	116.03	70.05	30.00	12.00
ESI(+)	Tyrosine	182.10	136.09	26.00	14.00
ESI(+)	Taurine	125.84	107.83	40.00	10.00
ESI(+)	Cysteine	122.05	76.02	28.00	12.00
ESI(+)	Alanine	90.02	44.09	24.00	6.00
ESI(+)	trans-4-Hydroxy-L-Proline	132.09	86.05	24.00	12.00
ESI(+)	Glycine	76.06	30.03	26.00	10.00
ESI(+)	Homoserine	120.03	74.00	30.00	10.00
ESI(+)	Threonine	120.03	74.00	24.00	8.00
ESI(+)	Glutamine	148.02	84.03	24.00	14.00
ESI(+)	Aspartic acid	134.07	74.00	26.00	12.00
ESI(+)	Glutamic acid	148.08	84.03	30.00	14.00
ESI(+)	Serine	106.01	60.02	18.00	8.00
ESI(+)	Asparagine	133.06	87.08	22.00	8.00
ESI(+)	Citrulline	176.13	70.03	14.00	18.00
ESI(+)	Arginine	175.08	70.09	42.00	20.00
ESI(+)	Histidine	156.10	110.09	14.00	14.00
ESI(+)	Lysine	147.08	84.02	20.00	14.00
ESI(+)	Ornithine	133.07	70.04	20.00	12.00

**Figure 1:**
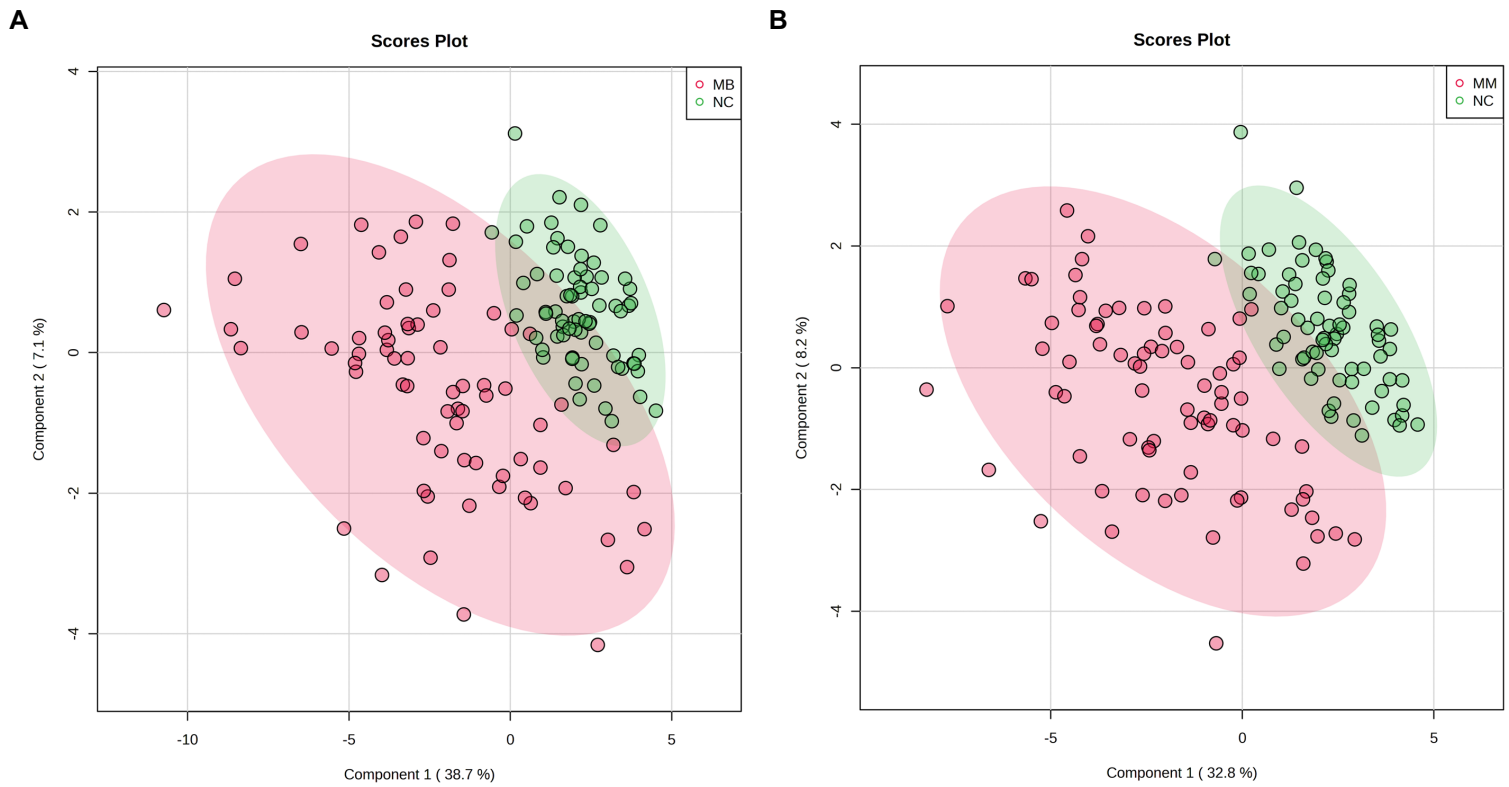
The score plots for PLS-DA analysis model of metabolomics data in the control, MB, and MM groups. **(A)**: PLS-DA results in the control and MB groups; **(B)** PLS-DA results in the control and MM group.

We adopted a PLS-DA approach to explore the potential metabolic differences in AAs between the MB, MM and NC. First, a permutation test revealed that the B/W ratio of the original classes (red arrow) different significantly from the permuted data distribution, which was consistent with reliable cross-validation (Supplementary Figure S1). Therefore, no overfitting was found according to the results of the permutation test. As observed on the PLS-DA score plot in Figure 1, the control patients exhibited a significantly distinct AAs profile, whereas that of the MB and MM groups overlapped (Supplementary Figure S2). However, good separation was achieved between the NC and MB or MM.

### Differentially abundant patterns of AAs

**Figure 2:**
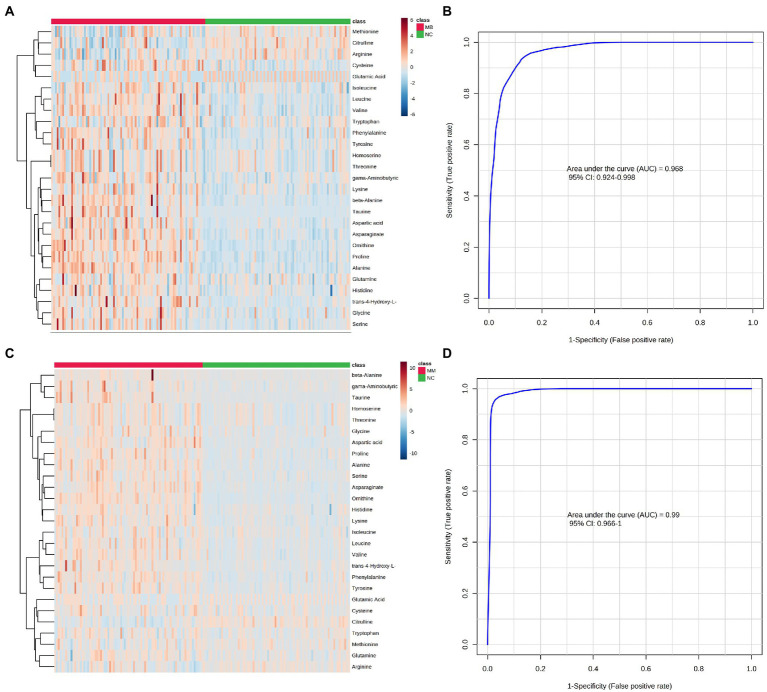
Differential expression of AAs concentration between the MB, MM and NC groups were plotted as heatmap (**A**: MB vs. NC; **C**: MM vs. NC). Red represents a higher concentration, but blue represents a lower concentration in heatmap. The performance of AAs biomarkers in distinguishing NC, MB, and MM groups. (**B**) The ROC curve of 5 AAs (ornithine, asparagine, valine, citrulline, and cysteine) diagnostic panel between the MB and NC group. (**D**) The ROC curve of 5 AAs (ornithine, taurine, phenylalanine, citrulline, and cysteine) diagnostic panel between the MM and NC group.

A univariate statistical analysis (FDR-corrected *p*-value <0.05) was used to evaluate and confirm the significant changes in AAs between the MM, MB, and NC. There were 26 significantly changed variables in MB comparing to NC (*p* < 0.05). The relative levels of these AAs can be observed on the heat map (Figure 2). The trends of altered AAs in MM, MB, and NC have been shown in Table 3. Moreover, upon comparing to NC, the levels of citrulline, arginine, and methionine were lower in the MB group, whereas most of altered AAs were significantly higher in the MB group. The changed AAs in MM group upon comparison to NC exhibited similar pattern with MB (Table 3). There were three AAs, including proline, asparagine, and cysteine, which were significantly changed in both MM and MB groups. Among them, proline and cysteine showed higher level in MM, whereas asparagine was elevated in MB (Supplementary Figure S3).

**Table 3:** Significantly altered amino acids in the different groups.

Amino acids	Trend		
	MB vs. NC	MM vs. NC	MB vs. MM
Ornithine	△↑	△↑	—
Asparagine	△↑	△↑	#↓
Alanine	△↑	△↑	—
Aspartic acid	△↑	△↑	—
Phenylalanine	△↑	△↑	—
Valine	△↑	△↑	—
Lysine	△↑	△↑	—
Citrulline	△↓	△↓	—
Serine	△↑	△↑	—
Proline	△↑	△↑	#↑
Taurine	△↑	△↑	—
Leucine	△↑	△↑	—
Histidine	△↑	△↑	—
Tyrosine	△↑	△↑	—
trans-4-Hydroxy-L-Proline	△↑	△↑	—
Glycine	△↑	△↑	—
γ-Aminobutyric acid	△↑	△↑	—
Homoserine	#↑	△↑	—
Threonine	#↑	△↑	—
β-Alanine	△↑	△↑	—
Isoleucine	△↑	△↑	—
Glutamine	#↑	△↑	—
Arginine	△↓	#↓	—
Cysteine	△↑	—	#↑
Tryptophan	#↑	#↑	—
Methionine	#↓	—	—

“↑” means a higher level of metabolites; “↓” means a lower level of metabolites; “△” means *P*<0.001; “#” means *P*<0.05, p value via FDR-corrected “— “represents no statistically significant difference.

### Establishment of diagnostic models for MB and MM

**Figure 3:**
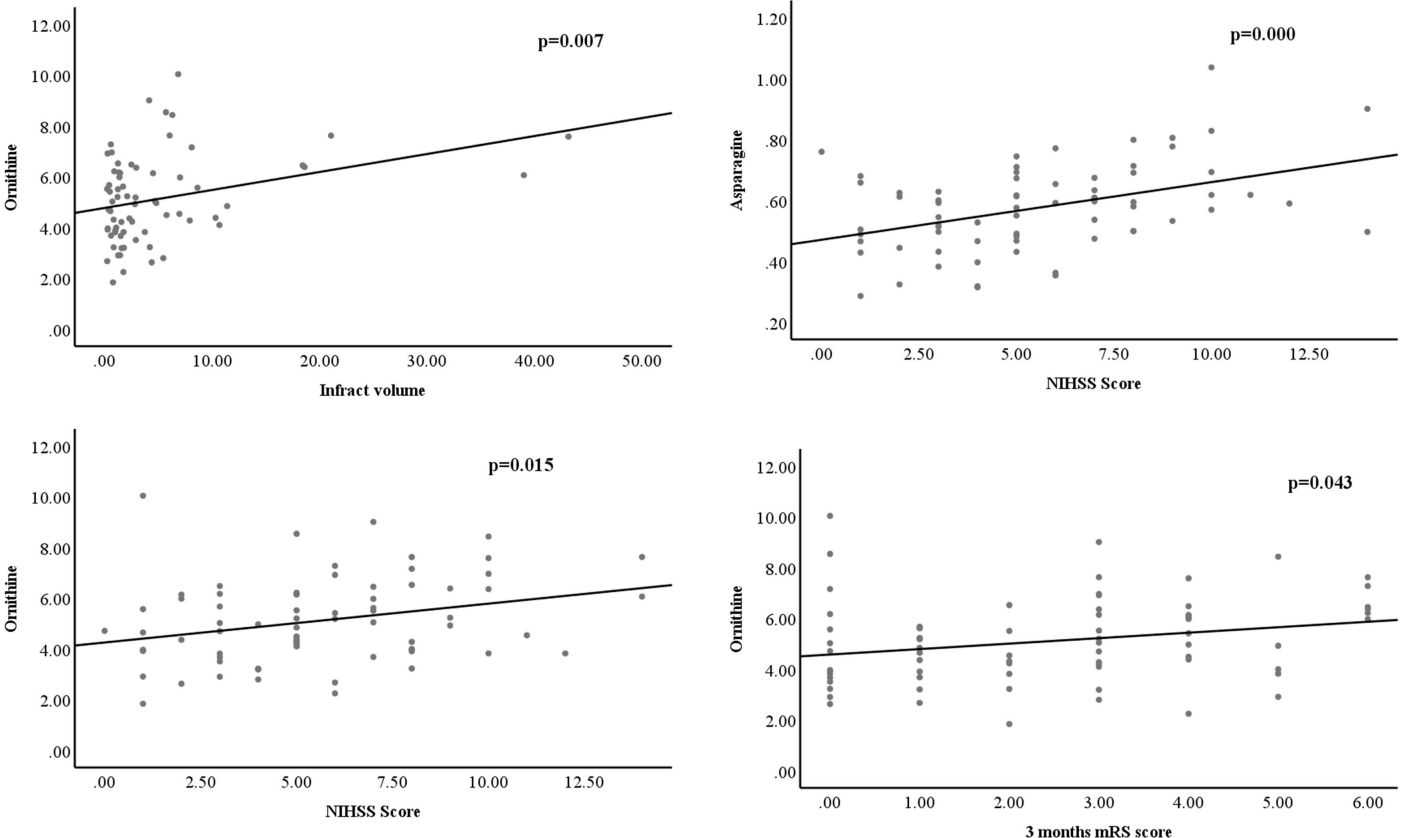
levels of ornithine were positively associated with infract volume, 3 months mRS score, and NIHSS score in MB, asparagine was positively associated with NIHSS score.

Based on all the significantly changed metabolites as described above, we established the diagnostic models for MB and MM. ROC curves were then used to assess the potential value of these metabolites as MB biomarkers in Metaboanalyst. To avoid overfitting, distinct features were selected based on overall ranks (AUC and T-statistic), K-means (KM) clustering, and LASSO algorithm. Thus, five optimal AAs including ornithine, asparagine, valine, citrulline, and cysteine identified in a potential biomarker panel with an AUC of 0.968 (95% CI 0.924–0.998) to discriminate MB patients from the control (Figure 2B). The average accuracy based on 100 cross validations was 0.907. We randomly selected 15 MB patients and 15 controls as hold out data and the accuracy for hold out data prediction was 1 (15/15) based on this model. Similarly, five different AAs, including ornithine, taurine, phenylalanine, citrulline, cysteine, yielded an AUC of 0.99 (95% CI 0.966–1) discriminate stroke mimic patients from the control (Figure 2D). In this model, the average accuracy based on 100 cross validations is 0.947 and the accuracy for hold out data prediction was 1 (15/15). Next, we found that levels of ornithine were positively associated with infract volume, 3 months mRS score, and NIHSS score in MB, asparagine was positively associated with NIHSS score (Figure 3). No significantly correlations were found between other potential biomarkers and infract volume, 3 months mRS score, and NIHSS score in MB (Supplementary Figures S4-S6).

### Pathway analysis

**Figure 4:**
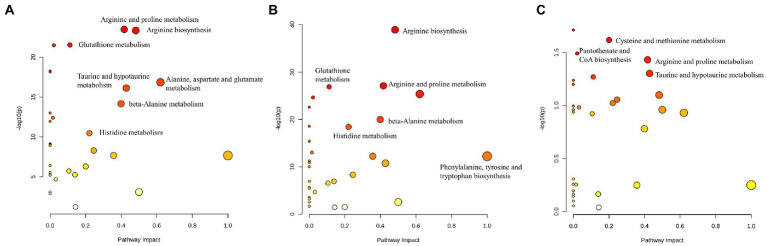
The control, MB, and MM groups were shown to differ significantly in several specific metabolic pathways. The influence on the relevant pathway was represented by node size, and the corresponding value of p from a pathway enrichment study was displayed by the node color. **(A)** MB vs. NC; **(B)** MM vs. NC; **(C)** MB vs. MM.

Pathway analysis performed by MetaboAnalyst was used to explore the potential pathways involved in regulating the levels of AAs. Those pathways that depicted low *p*-values and high pathway impacts have been shown in Figure 4. For MB and NC, different AAs were primarily associated with the following metabolic pathways: Arginine and proline metabolism, arginine biosynthesis, glutathione metabolism, pantothenate and CoA biosynthesis, alanine, aspartate and glutamate metabolism, taurine and hypotaurine metabolism, propanoate metabolism, and histidine metabolism (Figure 4A). However, For MM and control groups, similar metabolic pathways were obtained based on the pathway analysis (Figure 4B). There were 3disturbed metabolic pathways including cysteine and methionine metabolism, arginine and proline metabolism, and taurine and hypotaurine metabolism, for MM and MB groups (Figure 4C). The complete details of affected pathways can be observed in Table S1.

## Discussion

At present, the diagnosis of IS primarily depends on the medical history, physical examination, and imageological diagnosis, which are usually time-consuming and associated with low-specificity. Metabolomics has facilitated significant progress in understanding the mechanisms of metabolic reactions in the acute phase of IS, as well as in the identification of key metabolic pathways that are important for neuroprotection (25, 26). A number of altered metabolites have been reported, including organic acids, AAs, free fatty acids, lipids, and low-density lipoprotein in the serum and plasma of cerebral ischemia patients (8, 20, 27).

Among them, AAs are the most commonly identified metabolites after stroke and some of them could serve as high frequency metabolic biomarkers of IS. For example, glutamate plays an important role in mediating neuronal damage during cerebral ischemia. Glutamine, the main precursor of glutamate, was detected at significantly higher level in the plasma of patients with ischemic stroke. Phenylalanine is a derivative from glutamate and its metabolite is tyrosine. Increased phenylalanine level in IS patients has been found to be a compensatory response to the neurotoxic levels of glutamate. In addition, consistent with the previous studies (28, 29), we also found that glutamine, phenylalanine and tyrosine levels were increased significantly in MB patients (Table 3). Gamma-aminobutyric acid (GABA), an inhibitory neurotransmitter, derived from glutamate was also observed to be enhanced and its increase could be explained as a potential protective change in response to the excitatory neurotoxicity (30). Interestingly, we found that glutamate levels in IS patients were not altered compared to control in the current study, which was in contrast to the findings of the previous studies (28, 31). However, it has also been reported that serum concentrations of glutamate were normal in another study (32). This observation was explained because glutamate cannot easily cross the blood–brain barrier and affect the plasma levels. Overall, these findings related to alteration of glutamate levels in cerebral ischemia are contradictory and need further study.

BCAAs, including valine, leucine, and isoleucine, were also observed increased in MB related to control patients in current study. The various stages of stroke are linked to BCAA, which are essential in the regulation of glutamate/glutamine cycle and a significant source of glutamate nitrogen (33). They are significantly increased which may aid to explain the increased synthesis of neurotransmitters like glutamate and GABA. Interestingly, decreased valine and isoleucine have been observed in IS as well (28, 32). Interestingly, the differential changes in circulating BCAA may relate to the nutritional condition of IS patients in response to anabolic and catabolic alterations or metabolic comorbidities, such as diabetes, hypertension, hypercholesterolemia, or cardiovascular disease (34). Ornithine and citrulline, two metabolites linked to the arginine production pathway, can also exhibit dysregulation in IS patients. Nitric oxide (NO), a well-known endogenous vasodilator, is biosynthesized from arginine as a substrate. The elevated amount of Arg during MB in this study could be result of an amplified NO production that can receive input from the anaerobic condition of the brain.

Moreover, based on our PLS-DA result and heatmap (Figure 1, 2), we found that different AAs profile between NC and MB, while MM and MB were overlapped. This finding suggested that MM and MB shared similar AAs pattern that was also consistent with univariate analysis (Table 3). Next, the altered AAs related pathways in MB and MM were also explored. According to a previous review (20), it has been concluded that the most altered metabolic pathways in IS were those of arginine biosynthesis, alanine, aspartate and glutamate metabolism, aminoacyl-tRNA biosynthesis, and citrate cycle. Among them, several pathways were highly affected, including those of alanine, aspartate, and glutamate metabolism with high impact value. In our study, we found similar results that arginine and proline metabolism, arginine biosynthesis, glutathione metabolism, alanine, aspartate and glutamate metabolism, taurine and hypotaurine metabolism, and histidine metabolism were most significantly affected pathways in MB (Figure 4A).

In this study, the diagnostic model constructed by the 5 AAs (ornithine, asparagine, valine, citrulline, and cysteine) in MB showed good diagnostic performance based on overall ranks (AUC and T-statistic), K-means (KM) clustering, and LASSO algorithm, and showed an AUC of 0.968 (95% CI 0.924–0.998). However, since both the mild to moderate stroke patients were enrolled in the study, the sensitivity and specificity of this potential biomarker panel could be acceptable for early diagnosis of MB. Similarly, another 5 AAs panel was selected to separate MM from NC with an AUC of 0.99 (95% CI 0.966–1). From overall AAs profile, it was hard to distinguish between MB and MM. However, there were three AAs, including asparagine, cysteine, and proline, as well as three pathways including that of cysteine and methionine metabolism, arginine and proline metabolism, and taurine and hypotaurine metabolism, that were found to be significantly altered between MM and MB groups. These findings indicated that AAs could be possible biomarkers for early diagnosis of MB and MM patients. Moreover, levels of ornithine were positively associated with infract volume, 3 months mRS score, and NIHSS score in MB, asparagine was positively associated with NIHSS score (Figure 3). It indicated that ornithine is a very important metabolite for diagnosis and progression of MB and may offer prognostic value in MB patients. Another advantage of detecting AAs in this study is that they can be quantitative analyzed based on our established assay (35) and can be easily developed into a commercial kit for further clinical use.

Overall, it has been established that AA metabolism is essential for the onset and progression of IS, thereby making it a suitable target for therapeutic approaches. However, it is vital to incorporate other molecular biomarkers, such as lipids or proteins, which can significantly improve the sensitivity as well as specificity of the biomarkers pattern, and a multicenter experiment with large size samples is necessary for validation. We anticipate that these molecular markers can have enormous potential for additional clinical uses in the future.

## Conclusion

In this study, we have profiled different serum AAs of mild to moderate stroke patients, stroke mimic patients and healthy controls using targeted AA metabolomics. 27 AAs were quantitatively detected in serum of MB, MM and controls. Multivariate and univariate statistical analyses showed distinct AA profiles in MB, MM and NC. MB and MM showed similar AAs pattern based on the pathway analysis. In addition, we have constructed a diagnostic panel using a machine learning algorithm consisting of five AA molecules with good diagnostic efficiency for both MB and MM. Therefore, metabolism of AAs can be potentially considered as a key indicator for the prevention and treatment of IS.

## Data availability statement

The original contributions presented in the study are included in the article/supplementary files, further inquiries can be directed to the corresponding authors.

## Ethics statement

The studies involving human participants were reviewed and approved by the ethics committee from Liaocheng People’s Hospital. The patients/participants provided their written informed consent to participate in this study.

## Author contributions

ST and XX: conceptualization and validation. XuL and DY: methodology, funding acquisition, and supervision. XiL, FN, SW, and PZ: software. ST, XX, and DY: formal analysis. ST, XX, XiL, FH, PZ, and DG: data curation. ST and DY: writing the original draft preparation. ST, XX, and DY: writing, reviewing, and editing. ST: visualization. XuL: project administration. All authors have read and agreed to the published version of the manuscript.

## Funding

This research was funded by Science Foundation of Liaocheng People’s Hospital (no. 2018WS420), Medical and health technology development program in Shandong province (no. 202003070082) and Natural Science Foundation of Shandong Province (no. ZR2021MH215).

## Conflict of interest

The authors declare that the research was conducted in the absence of any commercial or financial relationships that could be construed as a potential conflict of interest.

## Publisher’s note

All claims expressed in this article are solely those of the authors and do not necessarily represent those of their affiliated organizations, or those of the publisher, the editors and the reviewers. Any product that may be evaluated in this article, or claim that may be made by its manufacturer, is not guaranteed or endorsed by the publisher.
