# Supplementary material for: Targeted metabolomics reveals serum changes of amino acids in mild to moderate ischemic stroke and stroke mimics
Source: Front Neurol. 2023 Apr 14;14:1153193. doi: 10.3389/fneur.2023.1153193 (PMC10140586; doi:10.3389/fneur.2023.1153193)
Supplement: Supplementary file 1 [file Data_Sheet_1.docx]

**Targeted metabolomics reveals serum changes of amino acids in mild to moderate ischemic stroke and stroke mimics**

**Materials and Methods**

**UPLC‑MS/MS**

The LC-MS/MS system consisted of a Waters ACQUITY UPLC system connected to a Waters (Milford, MA, USA) Xevo TQ-S triple quadrupole MS with a flow through-needle sample manager, a cooling autosampler, a column oven with temperature control, a degasser, and a binary pump. The chromatographic separations were performed at 40℃ by using a BEH C18 analytical column coupled with a UPLC column inline stainless-steel filter kit (0.2 μm filter). The mobile phase consisted of 2 mM ammonium formate in acetonitrile containing 0.2% formic acid as (A) and 1 mM ammonium formate in water containing 0.1% formic acid as (B) for 12 min. The following binary gradient was used for analysis: 0.0- 0.5 min (85-85% A), 0.5-5.5 min (85-80% A), 5.5-12.5 min (80-60% A), 12.5-13 min (60-85% A) at a flow rate of 0.3 mL/min. The strong and weak solutions used to wash the auto-sampler were acetonitrile/water (10:90, v/v) and acetonitrile/water (90:10, v/v) respectively. The UPLC eluate was then introduced into the Waters Xevo TQ-S system, a triple-quadrupole mass spectrometer equipped with an ESI interface, for the quantification of the analytes in positive ion mode (ESI+). The detection was conducted in multiple reaction monitoring (MRM) mode. The precursor-to-product ion pair, cone voltage (CV) and collision energy (CE) for each analyte have been shown in Table 2. The ESI-MS/MS parameters used were as follows: capillary voltage of 0.4 kV, desolvation temperature of 400℃, desolvation gas flow rate of 800 L/h (N2, 99.9% purity). Argon (99.999% purity) was introduced as the collision gas into the collision cell at a flow rate of 0.1 mL/min. The data acquisition was conducted by Masslynx 4.1 software and processed by TargetLynx (Waters, Milford, MA, USA).

**TABLE S1**︱ List of detailed information of changed pathways among three groups based on metabolic pathway analysis

|  | **Pathway** | **FDR** | **Impact** |
| --- | --- | --- | --- |
| MB vs Control | Alanine, aspartate and glutamate metabolism | 6.3026E-17 | 0.53446 |
|  | Arginine biosynthesis | 7.0802E-23 | 0.48223 |
|  | Taurine and hypotaurine metabolism | 2.97E-16 | 0.42857 |
|  | Arginine and proline metabolism | 7.0802E-23 | 0.39328 |
|  | beta-Alanine metabolism | 2.46E-14 | 0.39925 |
|  | Histidine metabolism | 8.34E-11 | 0.22131 |
|  | Glutathione metabolism | 2.5361E-21 | 0.11182 |
| MM vs control | Phenylalanine, tyrosine and tryptophan biosynthesis | 1.43E-12 | 1 |
|  | Alanine, aspartate and glutamate metabolism | 3.4446E-25 | 0.621 |
|  | Arginine biosynthesis | 3.9165E-38 | 0.48223 |
|  | Arginine and proline metabolism | 1.2549E-26 | 0.41713 |
|  | beta-Alanine metabolism | 2.68E-19 | 0.39925 |
|  | Histidine metabolism | 1.3413E-18 | 0.22131 |
|  | Glutathione metabolism | 1.3702E-26 | 0.11182 |
| MB vs MM | Taurine and hypotaurine metabolism | 0.04234 | 0.42857 |
|  | Arginine and proline metabolism | 0.037087 | 0.41713 |
|  | Cysteine and methionine metabolism | 0.02417 | 0.20038 |
|  |  |  |  |


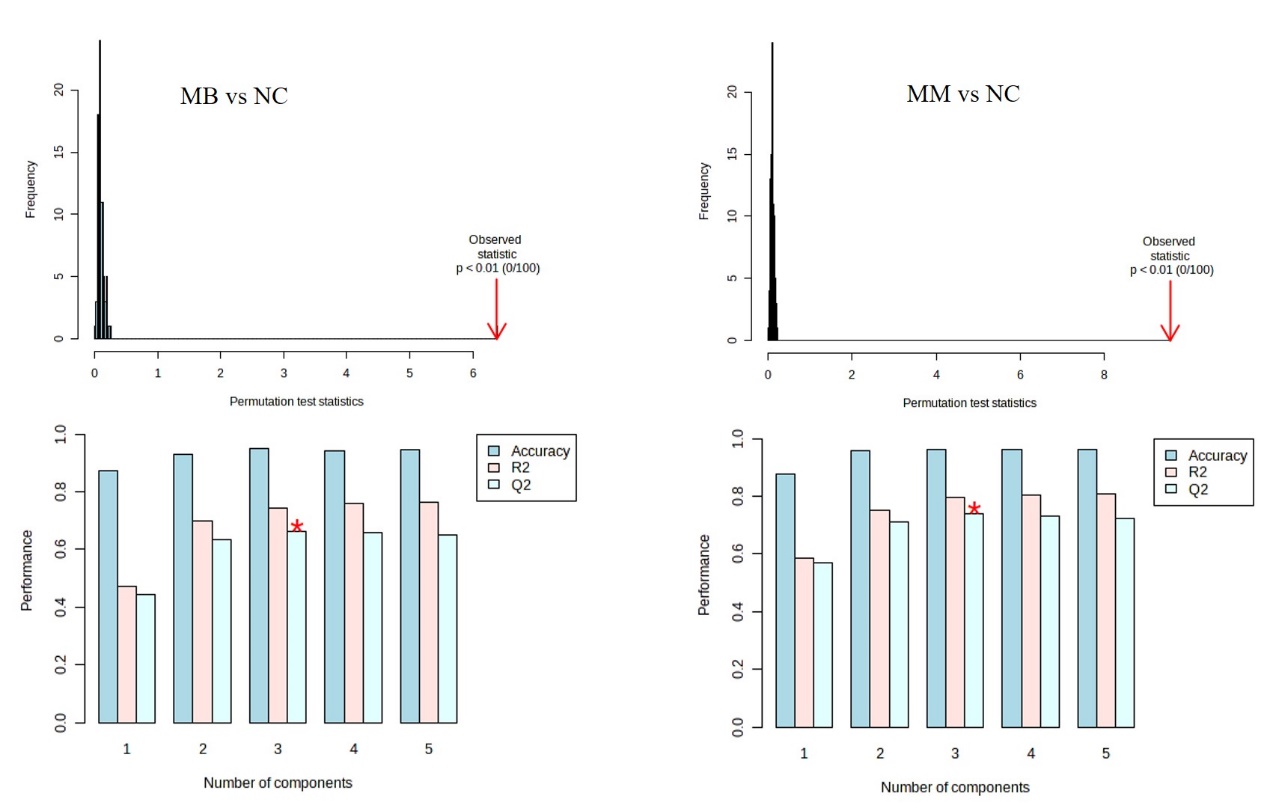


Figure S1 Validation of PLS-DA model using permutation test


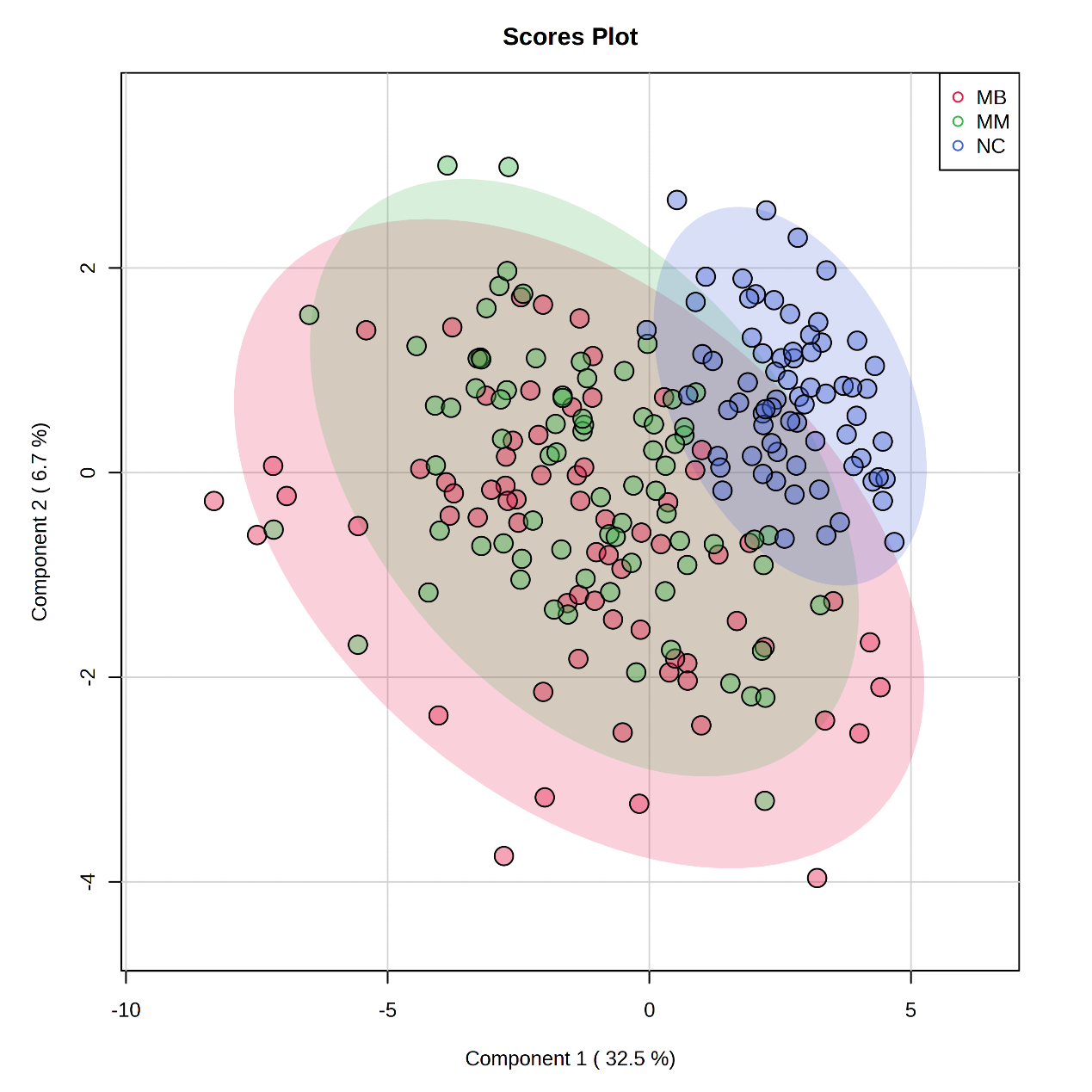


Figure S2 PLS-DA score plot of amino acids profile in three groups


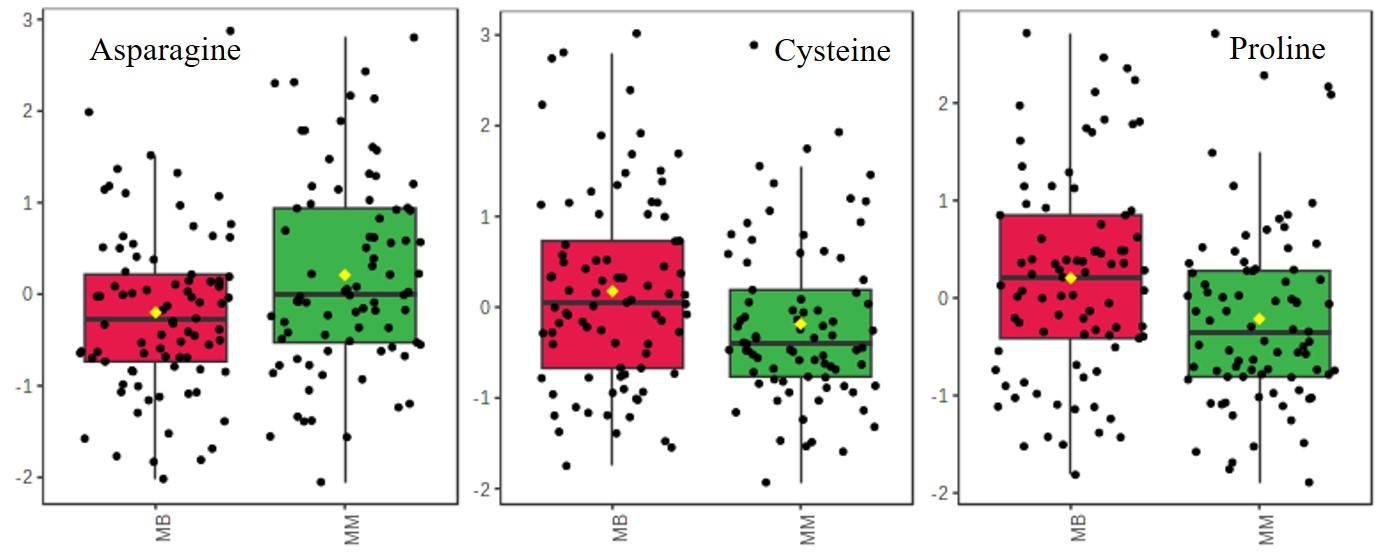


Figure S3 Box-plot of three changed amino acids in MB and MM group


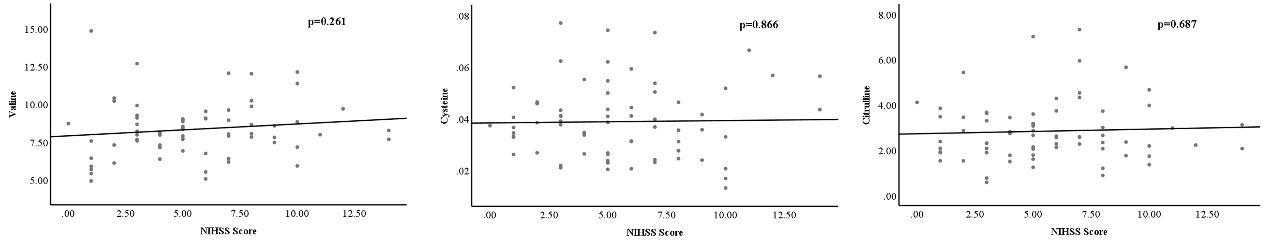


Figure S4 No significantly correlations were found between valine, citrulline, cysteine and NIHSS score in MB


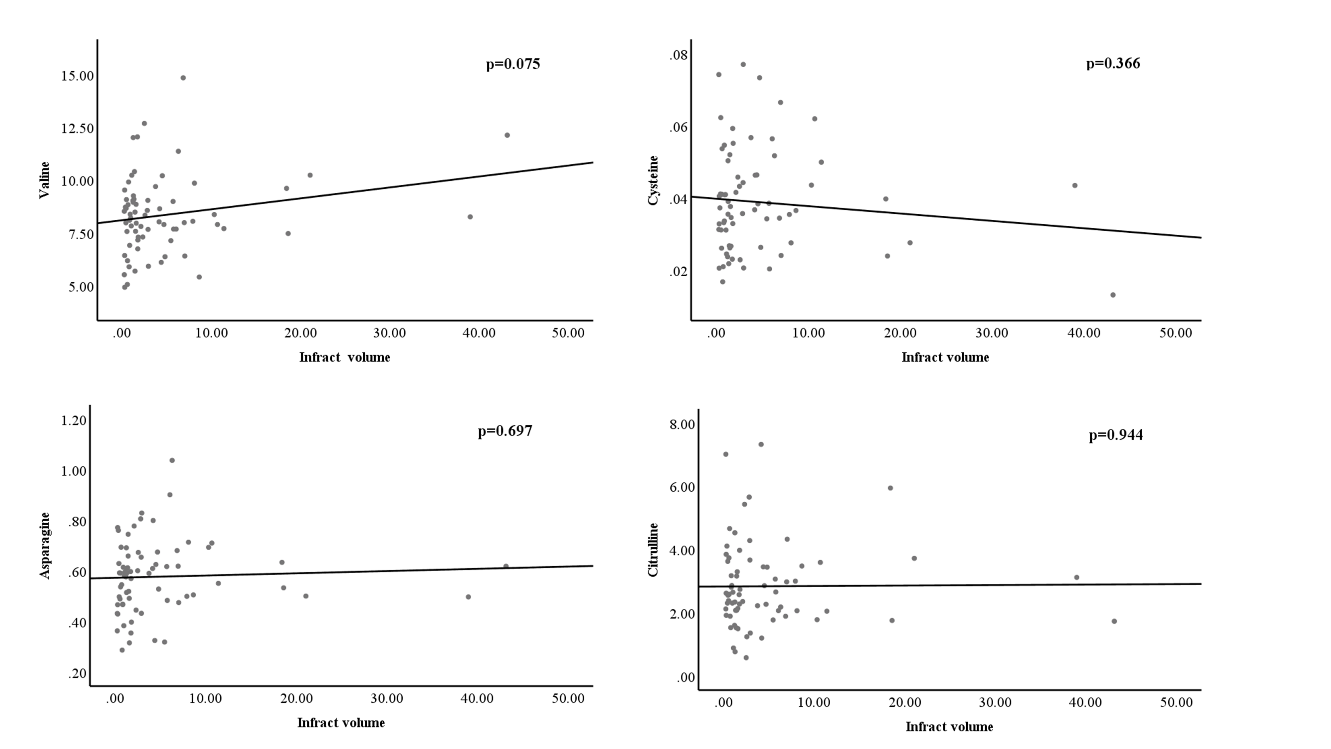


Figure S5 No significantly correlations were found between asparagine, valine, citrulline, and cysteine and infract volume in MB


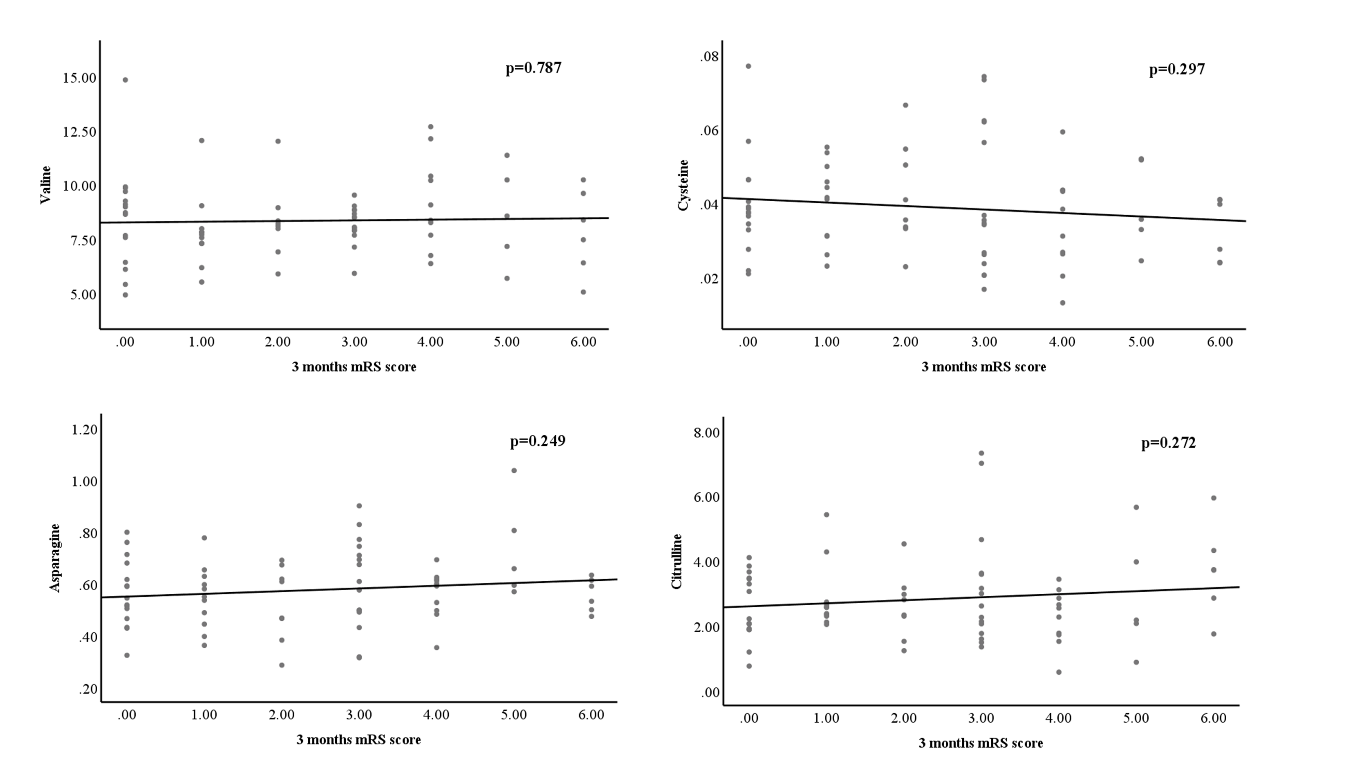


Figure S6 No significantly correlations were found between asparagine, valine, citrulline, and cysteine and 3 months mRS score in MB.
